# Isolated Left Main Coronary Artery Stenosis after Thoracic Radiation Therapy: To Operate or Not to Operate

**DOI:** 10.1155/2013/834164

**Published:** 2013-12-12

**Authors:** Osama Alsara, Ahmad Alsarah, Jagadeesh K. Kalavakunta, Heather Laird-Fick, George S. Abela

**Affiliations:** ^1^Michigan State University, Department of Internal Medicine, B-301 Clinical Center, East Lansing, MI 48824, USA; ^3^Michigan State University, Division of Cardiovascular Disease, B-208 Clinical Center, East Lansing, MI 48824, USA

## Abstract

Radiation therapy of neoplasms involving the chest or mediastinum results in a wide spectrum of cardiac complications including coronary artery disease, which can present in patients with few or no traditional cardiac risk factors. We report a case of radiation induced coronary artery disease in a 60-year-old female with a history of stage IIIA nonsmall cell lung carcinoma which was diagnosed eight years earlier and treated with chemotherapy and radiotherapy. She presented to the hospital with atypical chest pain that had occurred intermittently over the preceding week. Her initial electrocardiogram and cardiac enzymes were within normal limits. However, following an indeterminate exercise nuclear stress test, she developed chest pain and elevated cardiac enzymes. Coronary angiography demonstrated 90% stenosis of the left main coronary artery ostium, without any evidence of atherosclerotic disease or stenosis in other coronary arteries. She underwent surgical revascularization, which revealed dense adhesions surrounding the heart. During surgery, she developed severe bleeding and died. Coronary artery disease can present within years of radiation exposure, and ostial lesions are typical. Treatment is often challenging because of the effects of radiation on other tissues and the risks of revascularization procedures. Therefore, a multidisciplinary team approach should be considered.

## 1. Introduction

For the past few decades, radiation has been used effectively in the treatment of neoplasms involving the chest and mediastinum, including breast cancer, lymphomas, and lung cancer. However, radiotherapy of the chest can have a number of cardiac complications including pericarditis, pericardial effusions, conduction disorders, pancarditis, functional valvular defects, and coronary artery disease (CAD) [1]. CAD has been reported more commonly with Hodgkin's lymphoma, presumably because these patients tend to be younger and survive longer [2]. In this report we describe a 60-year-old woman with isolated left main coronary ostial stenosis diagnosed eight years after chest irradiation performed for stage IIIA nonsmall cell lung cancer. This case emphasizes the importance of clinical suspicion, prognostication, and the balancing risks and benefits of competing therapies.

## 2. Case Presentation

A 60-year-old woman with a history of lung cancer currently in remission was admitted with complaints of chest pain. She had recurrent substernal chest pain, increasing in severity, without radiation or associated symptoms during the preceding week. She denied any specific alleviating or provoking factors.

Past medical history was significant for hypertension and stage IIIA nonsmall cell lung carcinoma which was diagnosed eight years earlier. She had been treated with chemotherapy and radiotherapy. One year later she developed a malignant pericardial effusion, treated with a pericardial window, and recurrent pleural effusions, which were drained with pleural catheters. During the next six years, she was in full remission as confirmed by annual chest computed tomography (CT). Her medications included aspirin, metoprolol, iron, vitamin B12, and gefitinib. She was a nonsmoker and drinks alcohol occasionally.

**Figure 1:**
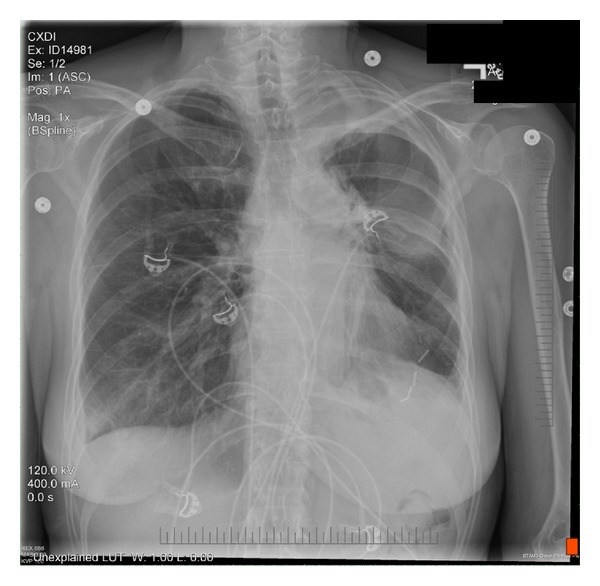
Posterior to anterior chest X-ray shows left perihilar parenchymal scars, surgical clips, and postoperative changes in the chest.

At presentation, her physical exam was benign. Initial cardiac biomarkers (creatine phosphokinase, troponin) and fasting lipid panel were within normal limits. The 12-lead electrocardiogram (ECG) showed normal sinus rhythm without any significant ST-T changes. Chest radiograph showed left perihilar parenchymal scars, surgical clips, and postoperative changes in the chest (Figure 1). Other laboratory studies, including two more sets of cardiac biomarkers, were normal.

**Figure 2:**
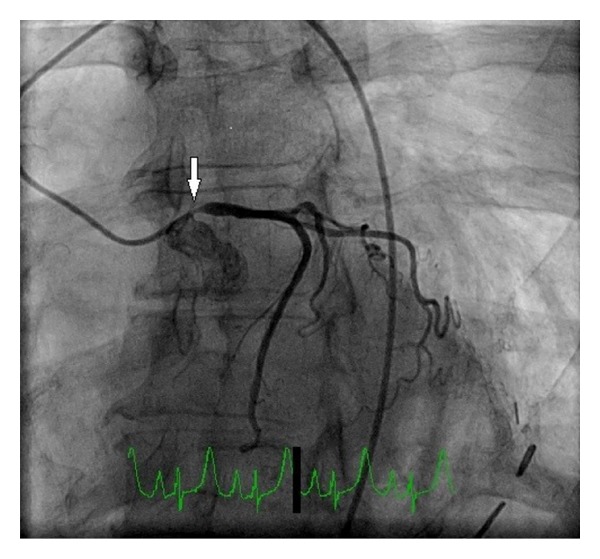
Coronary angiography with a right anterior oblique (RAO)/caudal projection. Arrow points to severe isolated ostial stenosis of left main coronary artery.

**Figure 3:**
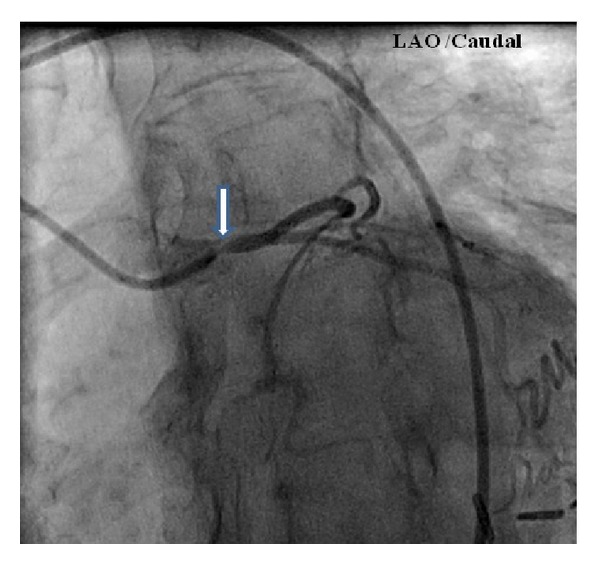
Coronary angiography of left main coronary artery in the left anterior oblique (LAO) and caudal view showing severe isolated ostial stenosis (arrow).

**Figure 4:**
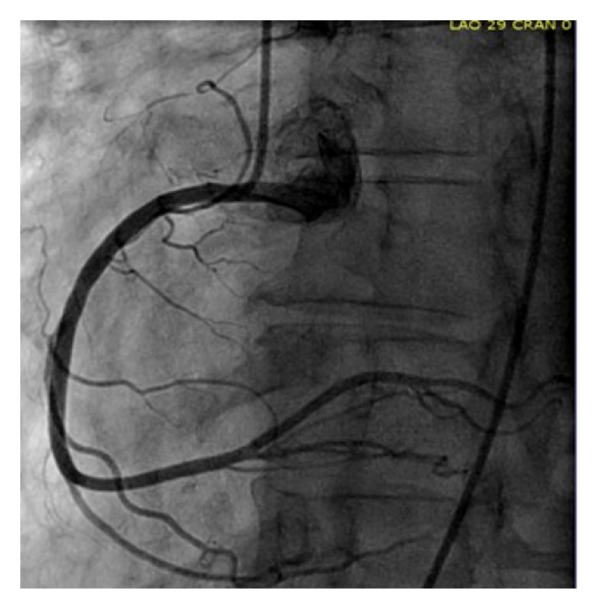
Coronary angiography of the right coronary artery in left anterior oblique (LAO) view.

She underwent an exercise nuclear stress test which demonstrated indeterminate electrocardiogram (ECG) changes with a small anteroseptal wall defect which appeared to be artifactual. Following the stress test, she developed retrosternal chest pain. Although her ECG did not show any acute changes, repeat serum troponin level was elevated at 0.42 ug/L. Coronary angiography was performed and demonstrated 90% stenosis of the left main coronary artery ostium, dominant right coronary artery, and no evidence of any atherosclerotic disease or stenosis in other coronary arteries (Figures 2, 3, and 4). Left ventricular ejection fraction was 55% without any regional wall motion abnormalities.

Syntax score was calculated to be 12% which predicts comparable outcomes of surgical and angioplastic procedures. Taking into consideration patient's history that may complicate the surgical treatment including her previous surgical interventions, radiation therapy, and chemical therapy, the interventional cardiology team recommended percutaneous coronary intervention (PCI) with stenting of the lesion. Cardiothoracic surgeons were consulted as well for further evaluation and they agreed that surgery will be challenging in this patient, but at the same time they felt that she may tolerate the surgery well, given her functional status, remission from cancer, and the low operative risk (EuroScore is 1.23%), and they suggested coronary artery bypass graft (CABG) surgery as a reasonable option due to the high failure rate of stenting ostial lesions and to avoid the possible cardiac arrest associated with the intervention on the stenotic ostium of left main coronary artery.

After discussing both treatment options, the risks and benefits of each procedure with the patient, she decided to undergo surgical revascularization. Overnight, she experienced excruciating anginal chest pain without any elevation in cardiac enzymes; as a result she was taken to surgery the next day.

Intraoperatively she was noted to have dense adhesions surrounding the heart. As a result, she developed severe bleeding from the adhesions. Despite aggressive transfusion of red blood cells and platelets, bleeding could not be controlled and she died during surgery. The family refused to allow autopsy to confirm the diagnosis.

## 3. Discussion

This is a case of a 60-year-old female with no classic cardiac risk factors, who developed a stenosis in the ostium of left main coronary artery eight years after undergoing radiation therapy for nonsmall cell lung cancer. She went to the operating room for CABG due to recurrent unstable angina but developed fatal bleeding related to side effects of both radiation and chemotherapy.

Thoracic irradiation can result in injury or inflammation of any of the cardiac structures. CAD has been reported in 5.5–12% of patients undergoing therapeutic chest radiotherapy, usually manifesting 3–30 years after radiation exposure [3]. Histology of coronary artery tissues obtained from affected patients have demonstrated intimal thickening with minimal extracellular lipid deposits [4]. Although the mechanism of radiation induced CAD (RICAD) is not fully understood, it is believed that radiation may stimulate cytokines and growth factors which in turn induce fibrosis and obstruction [5].

Ostial stenosis is typical of RICAD, since the vessels' openings are usually at the center of the radiation field. One study found that 16% of isolated coronary ostial stenosis was secondary to radiation therapy [6]. Isolated ostial stenosis of the left main coronary artery following radiation therapy has been reported in eight other cases in the literature [7–14] (Table 1). These patients were young to middle-aged females (mean age 40 years, range 26–51 years), with no or few traditional risk factors for CAD. Most presented with typical angina, between 2 and 20 years after radiotherapy. Our patient was older than those previously reported and developed symptoms eight years after therapy. Since RICAD presents about 16 years after radiation exposure and the 5 year survival life in patients with stage IIIA nonsmall cell lung cancer is estimated to be between 9 and 25%, it is very rare to see RICAD in this group [1, 15].

The strategy for revascularization of RICAD is typically the same as for atherosclerotic CAD [1]. Traditionally, CABG has been considered the treatment of choice for left main coronary artery disease (LMCAD) due to its role in increasing the life expectancy in patients with significant stenosis [16, 17]. The left internal mammary artery (LIMA) is typically used as a bypass conduit. Despite concerns that radiation might damage the LIMA, increase its fragility, and lead to early graft failure [18, 19], recent studies have documented patency rates comparable to that of venous grafts at 5 year follow-up [20, 21], making LIMA grafting a viable option for RICAD as well. Yet cardiac surgery in patients with past chest irradiation can still be technically challenging, with a greater risk of perioperative complications in the presence of pericardial thickening and retrosternal fibrosis. Although an early study has showed good outcomes of CABG treatments in patients with mediastinal radiation therapy, Wu et al. have found recently that patients with radiation induced heart disease have greater short-term and long-term mortality after cardiovascular surgeries compared to patients in same age and sex who underwent similar surgeries [22, 23]. This study also showed that the standard preoperative risk scores are usually suboptimal in predicting mortality of cardiac surgeries in patients with radiation heart disease [23]. With this in mind, other treatment options such as PCI should always be considered.

There is a growing body of literature supporting PCI with stenting of LMCAD due to atherosclerosis. Some trials have yielded rates of myocardial infarction and mortality similar to CABG, but with better perioperative outcomes [24]. As a result, PCI is now considered an acceptable alternative to surgical revascularization in some patients with LMCAD [16, 25, 26]. This approach could be particularly attractive for patients with radiation-induced adhesions. Ostial stents, though, have been associated with higher rates of restenosis related to their increased risk for proximal or distal displacement [27]. Several new techniques may improve the precision of ostial stent positioning [28], but long-term outcome data are not yet available.

Other comorbidities or medications may also influence the decision to choose percutaneous or surgical revascularization in patients with RICAD. For example, our patient was receiving the gefitinib, a chemotherapeutic agent which decreases platelet function via prostaglandin and thrombaxene and increases the bleeding risk [29]. There are no guidelines or studies on the perioperative management of patients receiving gefitinib. Its half-life is 48 hours, in contrast to 8 hours for the more common antiplatelet agents such as clopidogrel, so prolonged interruption of therapy may be necessary to decrease bleeding risk. We believe that additional preoperative evaluation by a hematologist-oncologist is warranted. Balancing the risk of bleeding against the risk of myocardial infarction or death becomes a complicated and imprecise calculus requiring the input of a multidisciplinary team.

Our case is unique, in that our patient was older at presentation, underwent chest irradiation for lung cancer rather than lymphoma, developed isolated left main coronary ostial stenosis, and her management was complicated by the combined effects of radiation and chemotherapy. More importantly, this case emphasizes the importance of a multidisciplinary team approach and participatory decision making when faced with significant risks of available treatment options.

## Figures and Tables

**Table 1:** Review of reported cases of isolated ostial stenosis of left main coronary artery following radiation therapy*.

Authors (year)	Gender	Age (Y)	RF	Neoplasm	Latency (Y)	Presenting symptoms
Radwaner et al. [7] (1987)	F	27	No	Hodgkin's lymphoma	8	Angina
Grollier et al. [8] (1988)	F	50	Smoking	Breast cancer	5	Angina
Orzan et al. [9] (1995)	F	26	No	Hodgkin's lymphoma	10	Dyspnea, pleural effusion
Takewa et al. [10] (1996)	F	45	/	Thymic carcinoid	3	Angina
Bensaid et al. [11] (1998)	F	43	No	Hodgkin's lymphoma	20	Myocardial infarction
Caus et al. [12] (1999)	F	47	Smoking	NH lymphoma	3	Angina
Victor and Parente [13] (2004)	F	51	Dyslipidemia	Hodgkin's lymphoma	2	Angina
Korosoglou et al. [14] (2012)	F	34	No	NH lymphoma	6	Angina

*F: female, Y: years, RF: risk factors for coronary artery disease, and NH: non-Hodgkin's.

The case of Takewa et al. [10] is in Japanese. The case of Bensaid et al. [11] is in French.
